# Relatively speaking

**DOI:** 10.1186/s12915-016-0265-2

**Published:** 2016-05-24

**Authors:** Graham Bell

**Affiliations:** BMC Biology, BioMed Central, 236 Gray’s Inn Road, London, WC1X 8HB UK

## Abstract

Protein X is a signalling molecule that stimulates apoptosis. Treatment of cells with Protein X results in five times higher levels of cell death than those seen in untreated cells (wild type), as measured by Caspase-positive cells. Based on previous work, the authors identify Protein Y as the putative receptor for Protein X and here try to test whether this is indeed the case. They claim that in a *geneY* mutant, where no receptor is expressed, treatment with Protein X no longer results in increased cell death, supporting the hypothesis that Protein Y is the receptor for signalling molecule X.

## Comment

A common way to represent data comparing two conditions is to plot the relative change of the outcome that is being measured by normalising both data sets to the average of the control condition. This gives the control values as ‘1’ and the experimental condition as a fold-change over the control.

**Fig. 1:**
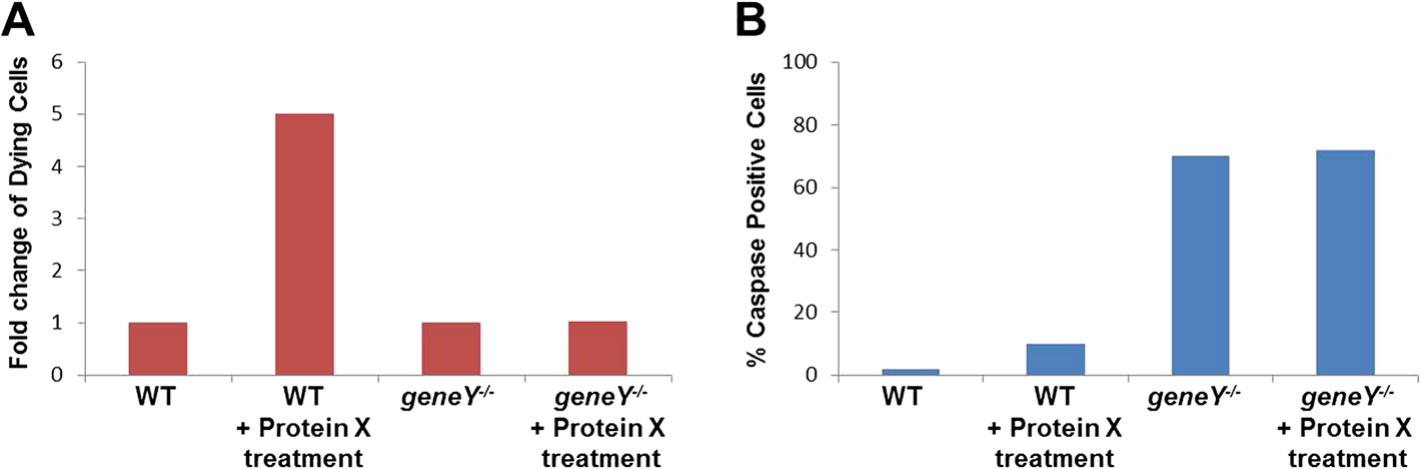
**a** Fold change of percentage of dying cells in wild-type (*WT*) and mutant samples, either treated with pro-apoptotic factor Protein X or untreated. For each genotype, cells treated with Protein X were compared with the respective untreated control. **b** Percentages of dying cells measured in wild-type and mutant samples, either treated with pro-apoptotic factor Protein X or untreated. Percentages are calculated as the number of Caspase-positive cells per 1000 cells

In this example, the authors show that in a wild-type (WT) background, treatment with Protein X caused a clear increase of dying cells when compared with the untreated control (Fig. 1a). Figure 1a also seems to show that there was no increase in cell death between mutant cells and mutant cells treated with Protein X.

However, plotting only the fold-change values can mask a more complicated story. Although in Fig. 1a the untreated WT and *geneY-/-* samples are both shown with values of 1, they do not represent the same actual numbers. The authors compared the treated cells of one genotype with untreated cells of the same genotype: thus, the two untreated conditions represent two separate controls—one the untreated WT, the other the untreated mutant—each normalised to 1. To see how this can mislead, we can look at Fig. 1b, where the actual percentages have been plotted.

Although treating cells with Protein X did not induce a noticeable increase in dying cells in the mutant background when represented as in Fig. 1a, the untreated mutant cells already have a very high level of cell death (Fig. 1b). Even if Protein X acts through a completely separate pathway to Protein Y, the further small increase in cell death from Protein X’s effects might not be noticeable because most of the mutant cells are already dead.

